# Improving detention ponds for effective stormwater management and water quality enhancement under future climate change: a simulation study using the PCSWMM model

**DOI:** 10.1038/s41598-023-32556-x

**Published:** 2023-04-05

**Authors:** Yasir Abduljaleel, Ali Salem, Faraz ul Haq, Ahmed Awad, Mustapha Amiri

**Affiliations:** 1 Department of Civil and Environmental Engineering, Washington State University, Richland, WA 99354 USA; 2 Civil Engineering Department, Faculty of Engineering, Minia University, Minia, 61111 Egypt; 3 Doctoral School of Earth Sciences, University of Pécs, Ifjúság Útja 6, Pecs, 7624 Hungary; 4 Centre of Excellence in Water Resources Engineering, University of Engineering and Technology, Lahore, 54890 Pakistan; 5 Egyptian Ministry of Water Resources and Irrigation (MWRI), Giza, 11925 Egypt; 6 Geomatics and Soil Management Laboratory, Faculty of Arts and Humanities, Université Mohammed Premier Oujda, 60000 Oujda, Morocco

**Keywords:** Civil engineering, Environmental impact

## Abstract

Urban surfaces are often covered by impermeable materials such as concrete and asphalt which intensify urban runoff and pollutant concentration during storm events, and lead to the deterioration of the quality of surrounding water bodies. Detention ponds are used in urban stormwater management, providing two-fold benefits: flood risk reduction and pollution load minimization. This paper investigates the performance of nine proposed detention ponds (across the city of Renton, Washington, USA) under different climate change scenarios. First, a statistical model was developed to estimate the pollutant load for the current and future periods and to understand the effects of increased rainfall on stormwater runoff and pollutant loads. The Personal Computer Storm Water Management Model (PCSWMM) platform is employed to calibrate an urban drainage model for quantifying stormwater runoff and corresponding pollutant loads. The calibrated model was used to investigate the performance of the proposed nine (9) detention ponds under future climate scenarios of 100-year design storms, leading to identifying if they are likely to reduce stormwater discharge and pollutant loads. Results indicated significant increases in stormwater pollutants due to increases in rainfall from 2023 to 2050 compared to the historical period 2000–2014. We found that the performance of the proposed detention ponds in reducing stormwater pollutants varied depending on the size and location of the detention ponds. Simulations for the future indicated that the selected detention ponds are likely to reduce the concentrations (loads) of different water quality constituents such as ammonia (NH_3_), nitrogen dioxide (NO_2_), nitrate (NO_3_), total phosphate (TP), and suspended solids (SS) ranging from 18 to 86%, 35–70%, 36–65%, 26–91%, and 34–81%, respectively. The study concluded that detention ponds can be used as a reliable solution for reducing stormwater flows and pollutant loads under a warmer future climate and an effective adaptation option to combat climate change related challenges in urban stormwater management.

## Introduction

Climate change and urban growth pose new challenges to urban stormwater management and making the preservation of urban aquatic ecosystems more vulnerable. Over the last decade, floods, among other natural disasters, caused an average of 0.1% of total deaths worldwide. The rampant urban development and the expansion of impervious surfaces over time have been causing a significant reduction in the infiltration rates of stormwater into the soil^1–3^, which lead to the so-called ‘urban stream syndrome’, characterized by the flashier hydrographs, high risks of flooding, and high pollutant loading in storm runoff^4,5^. Furthermore, stormwater runoff and corresponding pollutant loads are likely to increase in the future due to higher frequency and intensity of rainfall^6^. Hence, the water bodies at the stormwater outfall are more vulnerable to high flood and pollutant loads compared to the natural conditions^4,7^. Rapid urbanization along with intense and frequent rainfall, can wash pollutants from urban surfaces at relatively higher rates and cause significant negative impacts on downstream receiving ecosystems^8^. To mitigate these effects, various stormwater management techniques are available for retaining or restoring the natural hydrologic functions of urbanized catchments and reducing pollutant loads in stormwater discharge, known as ‘Best Management Practices’ (BMPs)^9,10^.

Among the BMPs, low impact developments (LID) based stormwater management practices are the most prominent and extensively used in cities across the world, with frequent applications mainly focusing on assessing LID—hydrological performance and hydraulic behavior during floods^11–15^. Stormwater management practices based on LIDs have shifted the focus of urban stormwater drainage systems that use pipes for storage and infiltration-based systems^16–18^. The underlying fundamental principle of LID is to keep the post-development hydrological environment of a location as close to its natural state as possible^16,19^. However, it is challenging to assess the effectiveness of LIDs in terms of how they will keep the pre-development natural hydrologic states unchanged or near to unchanged even after urban development^20^. Therefore, various governmental agencies, including the United States Environmental Protection Agency (US EPA), developed guidelines for the effective implementation of LIDs using different numerical modeling approaches those help in evaluating their performance in reducing runoff, peak flows, and pollutant loads. Several numerical models such as Distributed Routing Rainfall-Runoff Model (DR3M)^21^, Long-Term Hydrologic Impact Assessment-Low Impact Development (L-THIA-LID)**,** Personal Computer Storm Water Management Model (PCSWMM)^22^ and Urban Drainage and Sewer Model (MOUSE) are developed by various research groups. These models are widely used to assess pollution reduction in urban areas and can simulate the effects of LID on hydrological processes^17,18,23^. The Storm Water Management Model (SWMM) developed by US EPA is one of the most widely used numerical models in the United States, which readily simulates the performance of LIDs. Many researchers employed the SWMM model to investigate the stormwater discharge and pollutants concentration for urban catchments during a storm event and they reported that SWMM performs well in simulating storm runoff and pollutant concentration with reasonable performance.

LIDs, including detention pond, detention basins, and wetlands are centralized measures that treat stormwater runoff at the end of a catchment or drainage area^24^, whereas green roofs, bioswales, pervious pavements are employed to manage stormwater at source^25^. Detention ponds are utilized more frequently than other LIDs because of their conventional and straightforward concepts, as well as their low costs and minimal negative effects on the environment^26,27^. The main operational strategy behind detention ponds is to mitigate downstream flood risks and water pollution by temporarily storing stormwater in the basin before releasing it slowly over a prolonged period of time^8,14,28,29^. Thus, they help minimize sudden flood pulses and pollutant loads of downstream receiving water. Besides, detention ponds allow higher infiltration of stored stormwater leading groundwater recharge. Parts of King County in Washington have previously experienced water pollution problems due to untreated stormwater discharge^30^. The existing stormwater system includes pipes, storm drains, and gutters which collect stormwater from the watershed and re-route it to a nearby river or lake directly without treatment. Stormwater from roads, roofs, lawns, and ground surfaces wash off contaminants such as oil and grease, petroleum, pesticides, and fertilizers leading to water quality deterioration problems in King County^31^. Most of the existing stormwater systems in the King County are not designed to treat contaminated stormwater before discharging it into recipients. To minimize this problem, King County decided to remove pollutants and control flow rates of stormwater by incorporating a stormwater detention pond to store accumulated stormwater runoff and allow pollutants to settle^32^.

This study focuses on a watershed located in the city of Renton where flood susceptibility has been increased because of expanding impervious areas due to rapid urbanization^33^. Thus, the watershed is a reasonable selection to assess the suitability and performance of detention ponds in reducing flood risk and mitigating pollutant (nutrient and sediment) load from urban stormwater. Hence, this study focuses to investigate the performance of detention ponds in mitigating flood risk and pollutant loads from future storm water runoff using an urban watershed in the Renton City, Washington, USA, as an example. The storm water management model is calibrated for the study area in the PCSWMM platform using historical information and then adjusted to future precipitation obtained from several Regional Climate Models (RCM). The study will optimize the detention ponds based on their optimal spatial configurations within the watershed and considering multiple decision variables such as the number and volume of the detention ponds, combinations of different location of the detention ponds, as well as various implementation constrains (e.g., size and number of detention ponds in each sub-catchment) to reduce flood risks (i.e., reduction in total and peak flows) and minimize pollutant loads.

## Materials and methods

### Study area

**Figure 1:**
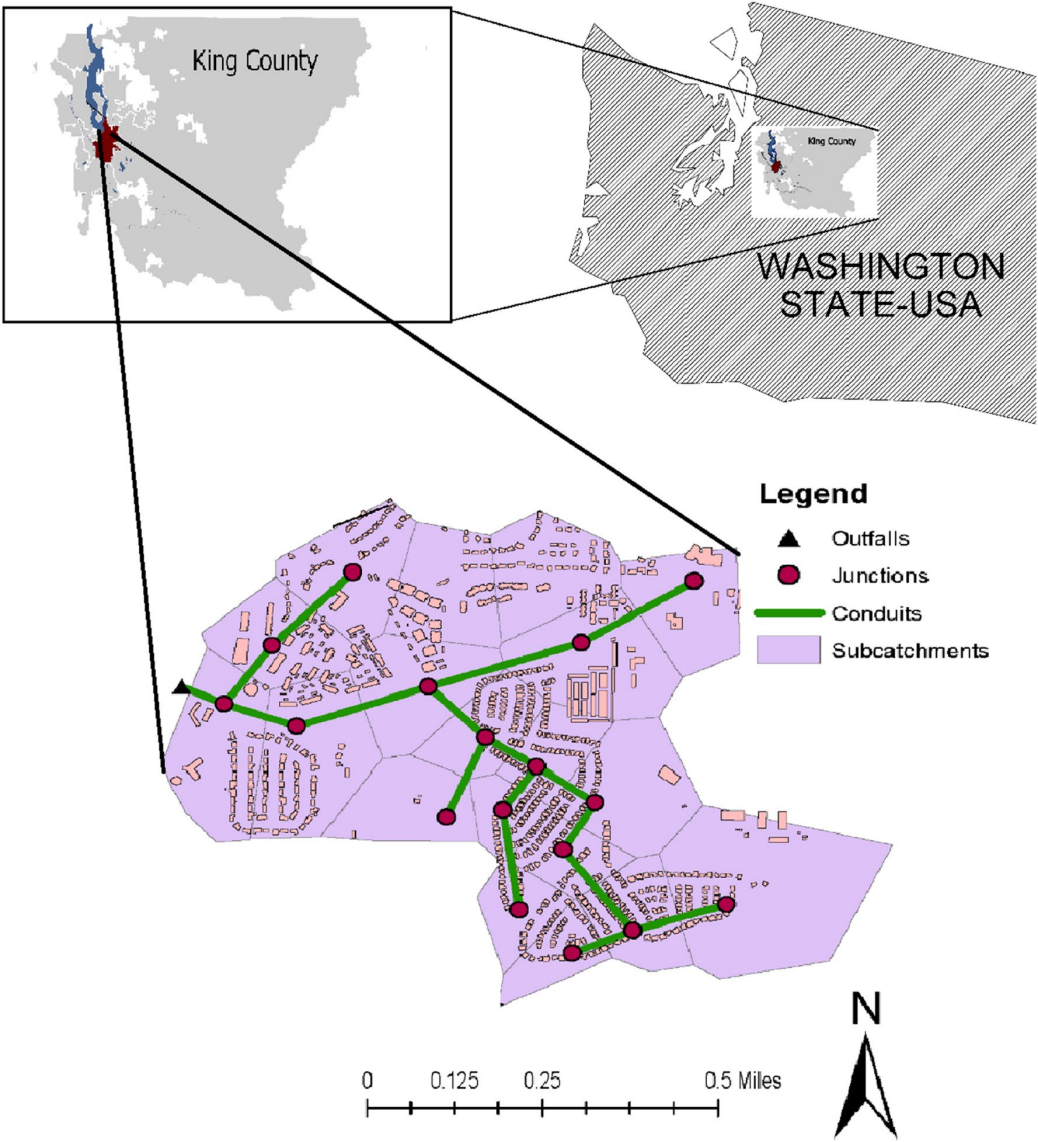
Location of the study area.

This study considers a watershed located in the city of Renton, Washington that covers an area of 12 km^2^ (Fig. 1). The watershed has a total impervious area of around 7.2 km^2^ includes residential area, commercial buildings, roadways, parking lots, and a paved area. The rest of the area is pervious and covered with woodland (15%) and short grass, pasture, and lawn (50%). The remaining 35% of the pervious area composed of open spaces and gravel roads. The study area has a relatively flat topography (slopes range from 1 to 5%) allows water to flow to the northwest by gravity. Stormwater is accumulated from the watershed and discharge into the adjacent streams (i.e., Lake Washington) through a underground pipe network that collects storm runoff from streets, parking zones, and buildings. The collected and accumulated stormwater is release into the northwest outfall of the study area (Fig. 1). In this study, we first investigated the capacity of the existing pond. We considered that the stormwater runoff will be directed to the stormwater treatment facilities (the proposed detention ponds) before discharging into the outfall. The study explored adjusted size of the proposed detention ponds to mitigate stormwater runoff and enhancing its quality. The proposed detention pond capacities were thought to be capable of conveying stormwater with a peak flow equivalent to a 100-year return level of a 24-h storm event for the historical and future periods (2023–2050) and prevent on-site flooding or erosion. According to the Flood Emergency Management Authority (FEMA), the proposed area mostly fall in Zones X and AE. The 100-year floodplain is located at an approximate elevation of 16 m in Zone AE; however, the FEMA 100-year floodplain elevation is 15–40 m (NAVD 88), and the compensatory elevation is at an elevation of 20.16 m (NAVD 88), and the existing site elevations for this study range from approximately 16–38 m. The selected study area has two regions with elevations less than 20.16 m, and for this purpose, the compensatory storage area shall be connected to the floodplain via an overflow spillway (elevation 16 m). In this case, the proposed stormwater detention pond provides a maximum storage below an elevation of 20.16 m (NAVD 88).

### Data sets

Data collection is an important and crucial task to better manage stormwater events^34^. The spatial and temporal datasets used in this study were as follows:

- Digital Elevation Model (DEM) (resolution: 5 m) provided by City of Renton
- Land use land cover (LULC) (resolution: 10 m) downloaded from Multi-Resolution Land Characteristics (MRLC).
- CGCM3-CRCM (Canadian Global Climate Model v.3–Canadian Regional Climate Model), CGCM3-RCM3, (Canadian Global Climate Model v.3–Regional Climate Model v.3) and GFDL-RCM3 (Geophysical Fluid Dynamics Laboratory GCM − Regional Climate Model v.3) are obtained from global climate model (North American Regional Climate Change Assessment Program (NARCCAP) (http://www.narccap.ucar.edu/data).
- Soil properties (resolution: 30 m acquired from Gridded SSURGO (SSURGO) from USDA-NRCS.
- Rainfall data at 15-min interval from January 1st, 2022, to December 31st, 2022, was collected from King County.

The DEM was used to delineate the watershed, to identify the flow directions and help design the stormwater conveyance system ^35^. Elevation and slope across the watershed were estimated from the 10-m resolution DEM. The DEM used in this study was provided by the Geospatial Data Gateway (GDG) of the Natural Resources Conservation Service (NRCS). The study area were divided into sub-basins based on the land slopes, LULC and soil types.

We followed the USDA Soil Conservation Service (1972) classification to classify the soil across the selected watershed, which consists of four major types (i.e., A, B, C, and D) depending on their infiltration capacity (Table 1). Sandy soils have higher infiltration rates that leads to relatively low runoff generation and hence a low runoff coefficient. On the other hand, soils with minimal infiltration rates (e.g., clay) have relatively high runoff rates and thus a high runoff coefficient. Loose soils (e.g., sandy soil) often found in low-lying areas produces insignificant runoff as compared to that from cohesive soils (e.g., clay).

**Table 1:** Soil groups as classified by USDA SCS.

Group	Infiltration rate (inches/h)		Soil texture
A	0.30–0.45		Deep sand, loamy sand, Deep Loess, Aggregated soils
B	0.15–0.30		Silt loam or loam, Shallow Loess
C	0.05–0.15		Clay Loams, sandy clay loam, low organic matter, high clay soils
D	0.00–0.05		Clay loam, silty clay loam (swell significantly when wet, heavy plastic soils)

**Figure 2:**
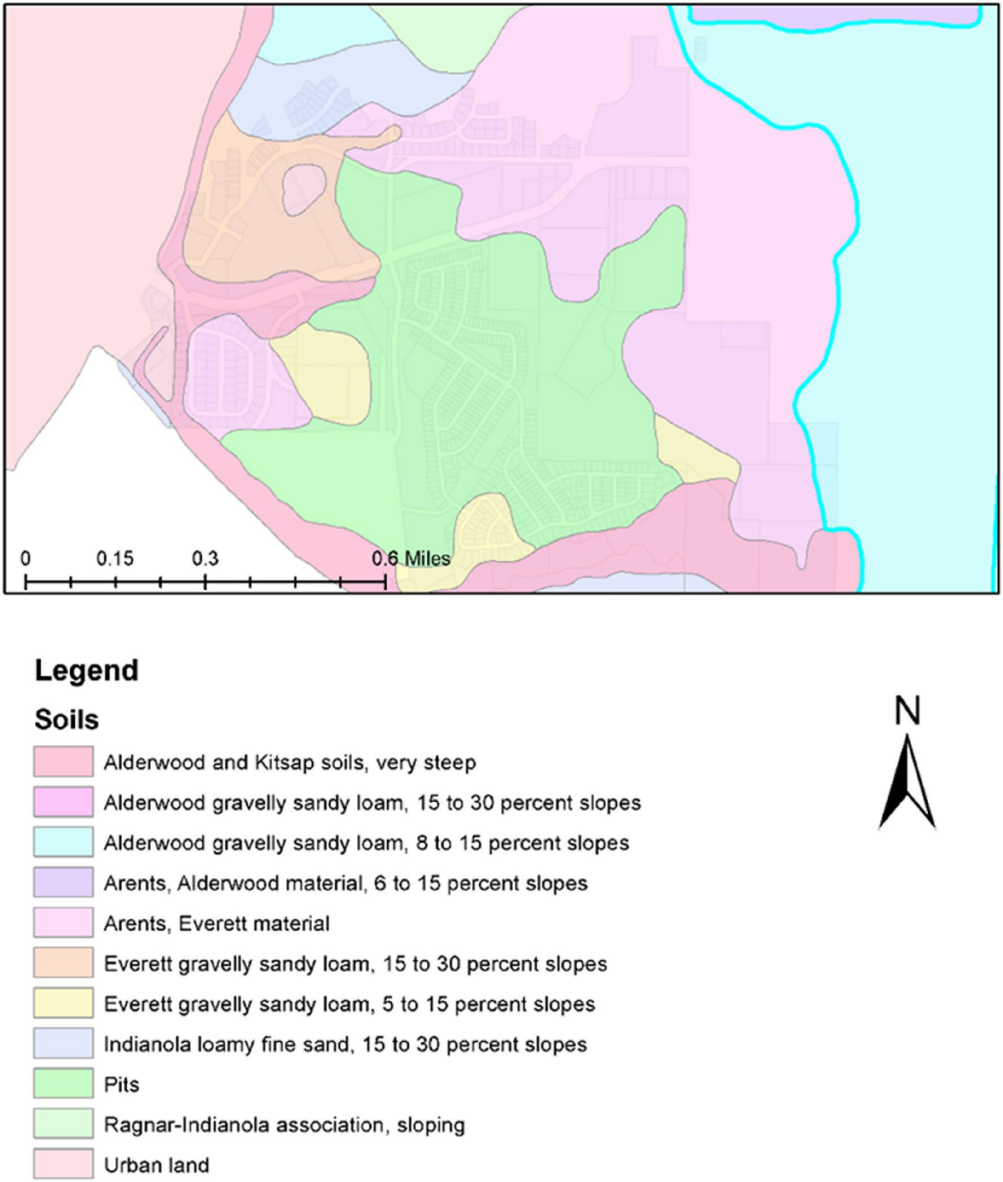
Distribution of different soil classes across the study area (Renton city watershed).

The study area is underlain by a fill soil layer (thickness between 0.5 and 1.5 m) with different textures (e.g., medium dense and medium to coarse silty sand). This fill layer is followed by an alluvial soil with interbedded layers of different texture (including soft silty clay to clayey silt, fine to coarse sand, silty fine sand, and very loose to medium dense silt). The basin soils were classified as AgC (Alderwood gravely, sandy loam, six to fifteen percent slopes), which fell under hydrologic group C of a slow infiltration rate when thoroughly wetted. Figure 2 shows the spatial distribution of different soil types across the study area.

**Figure 3:**
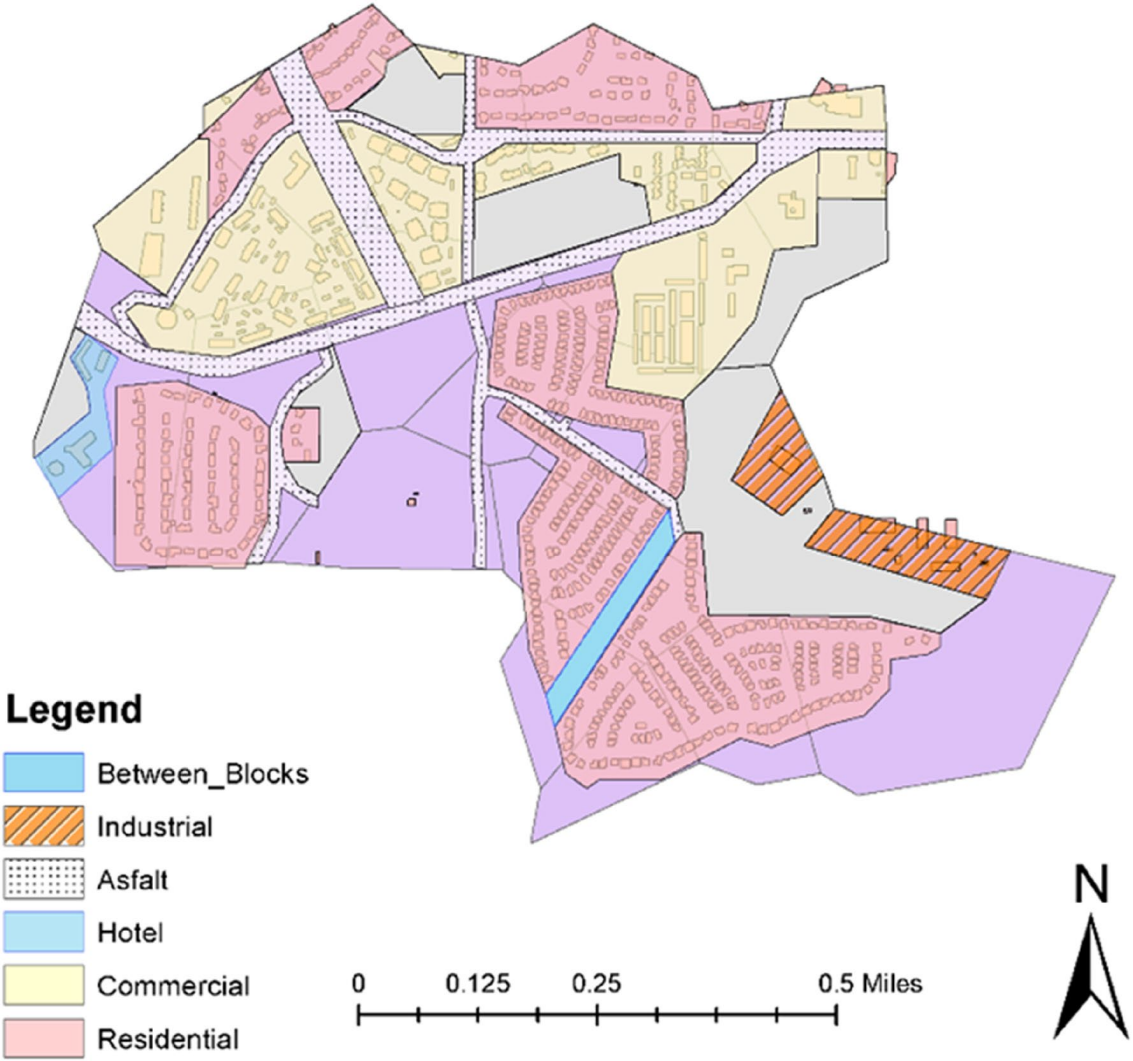
Land use/land cover (LULC) map of Renton city watershed.

The land use data for the study area is collected from satellite images, which are then categorized using Geographic Information System tool (GIS) (Fig. 3). The drainage boundary of the study area mainly includes residential and paved areas.

### Model development

We employed the widely used PCSWMM modelling platform to calibrate the stormwater (rainfall-runoff) model in the historical period and then adjusted with the storm from climate model output to quantify storm water flows (total and peak flows). PCSWMM uses the Curve Number (CN) approach for infiltration and dynamic wave routing by solving the one-dimensional St. Venant flow equations^36^. Given that the study focuses on a small watershed, the approach worked almost perfectly for runoff computations. The watershed was divided into 18 sub-catchments (see Fig. 1). The Curve Number method was selected due to its efficiency reported in many previous studies and the availability of required data. The model requires fewer parameters, yet it is stable compared to other available models such as the Green-Ampt and provides reliable estimation^37^. CNs were assigned to each land use category separately based on their soil types and finally a weighted CN was calculated for each sub-basin.

**Figure 4:**
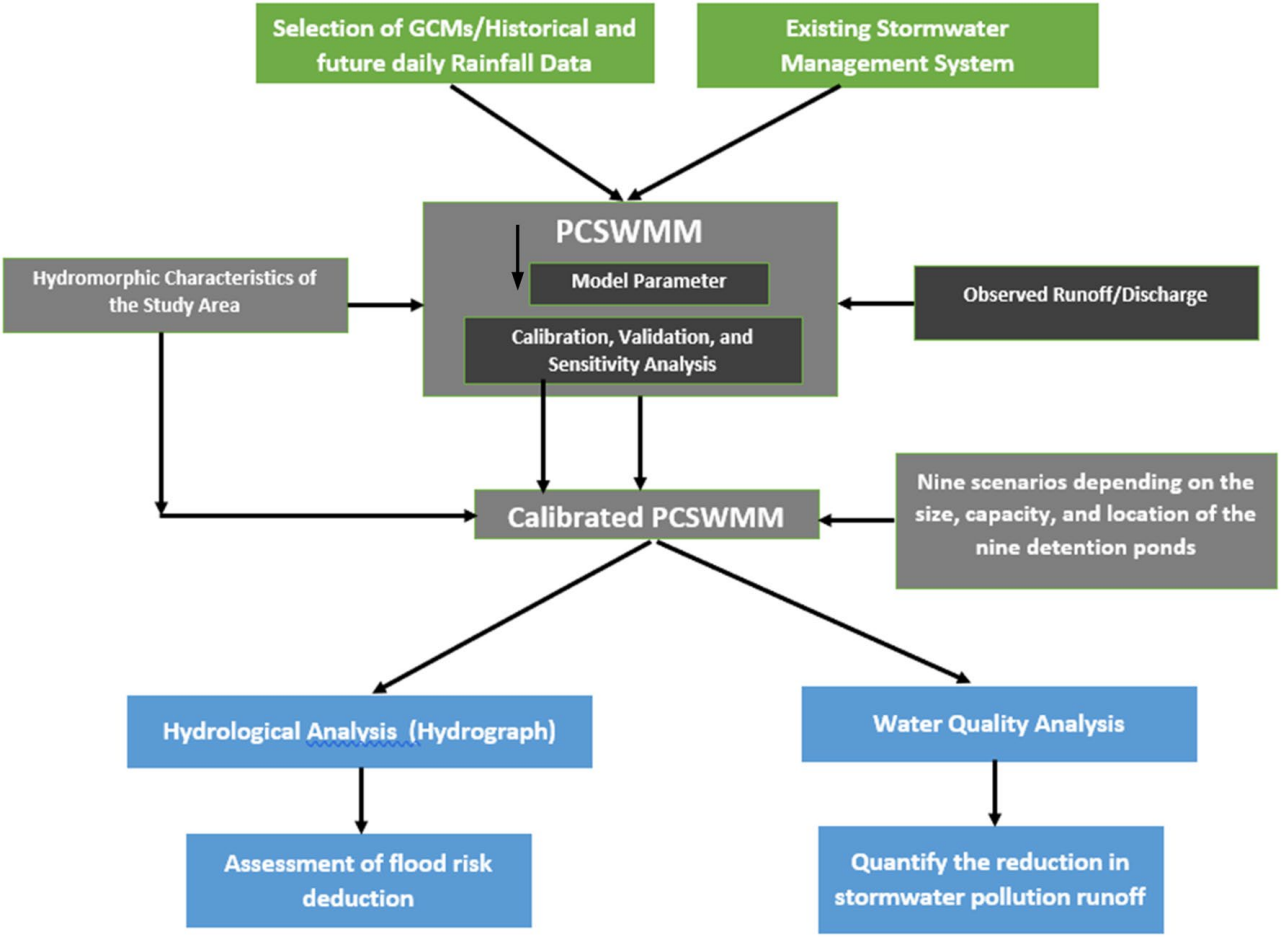
Flowchart showing the integrated framework of methodology.

The methodological framework adopted in the present study is shown in Fig. 4. The runoff volume (under historical and future scenario) is first simulated without considering any proposed detention ponds using the model validated within the PCSWMM modeling framework. Then, the model is adjusted for the rainfall (design storms) of different future climate change scenarios considering different combinations of detention ponds based on their volumes and locations were simulated to assess the performance of the detention ponds in mitigating future runoff and reducing pollutant loads. Finally, the size of the flow control outlet structure and detention pond facility were finalized and proposed for the simulated peak flows and pollutant loads over the future periods. Table 2 lists the parameters which were considered in setting up the models in PCSWMM.

**Table 2:** List of parameters considered for development of PCSWMM model.

Parameter/simulation options	Description		Value	Source
Infiltration Method	Describes how rainfall infiltrates to the upper zone of soil in a catchment		Curve Number	User defined
N Imperv	Surface roughness (Manning’s n) for overland flow of impervious portion of a sub-catchment		Various	SWMM User Manual, Appendix A6^31^
Zero Imperv	Fraction of the impervious area without depression storage		Various	SWMM User Manual (typical value)^31^
Width of Sub catchment	Width of sub catchment area for overland flow length of impervious		Various	SWMM User Manual (typical value)^31^
Routing Method	conveyance routes		Dynamic Wave	Classified by user
Storm event -type IA	NRCS Type 1A rainfall distribution		Various	Design storms of for historical and future return periods
Simulation period	Continuous simulation for 15 min time steps		Various	Based on rainfall distribution
Storage (underlying soils)	CN-model-Parameter		Various	^38^
Surface slope (%)	The slope of each sub catchment		Variable	DEM and GIS
Surface layer	Vegetation volume (fraction), % of a storage depth's volume that is taken up by vegetation		Variable	^33^

To calibrate the model in the PCSWMM platform, historical precipitation obtained from the North American Regional Climate Change Assessment Program (NARCCAP) (http://www.narccap.ucar.edu/data/ ) for the period spanning precipitation baseline as 2000 to 2014 were used. To simulate the climate change scenarios, daily rainfall obtained from the climate model (i.e., RCMs) simulations were first converted to hourly rainfall using standard methods such SCS Type II, NOAA Atlas 14 Nested, and Huff (first quartile) cumulative rainfall distribution. Then the information was used for developing the intensity–duration–frequency (IDF) curve for different climate scenarios. To develop IDF curves statistical frequency analysis was employed, whereby annual maximum rainfall series of a given duration is fitted to a best explaining extreme value distribution (e.g., Gumbel) to estimate the quantiles of rainfall for different return periods. Rainfall data from GFDL-ECP2 (Geophysical Fluid Dynamics Laboratory GCM—Experimental Climate Prediction Center), CGCM3-RCM3 (Canadian Global Climate Model v.3–Regional Climate Model v.3) and CGCM3-RCM3 (Canadian Global Climate Model v.3–Regional Climate Model v.3) climate models under different climate change scenarios are used to develop the corresponding rainfall intensity–duration–frequency (IDF) curves. In-situ observed meteorological data are used to develop IDF curves for the historical period (2000 – 2014).

The trained PCSWMM model is used to simulate stormwater runoff for different design storms obtained from the IDFs corresponding to selected climate scenarios. The model was equipped with different scenarios (will discuss in Sect. 2.4 in details) of detention ponds to examine their performance in reducing stormwater flow and pollutant load under warmer future climate. The pollutant load from the watershed was calculated from the annual total flow volume. The pollutant concentrations (C) provided by King County were used in conjunction with the total flow volume to estimate the Event Mean Concentration (EMC). Event means concentration (EMC) was expressed with a flow-weighted average concentration represented as the total pollutant mass divided by the total runoff volume. This can be computed as follows^39^:

1 $$EMC=\frac{\mathrm{M}}{\mathrm{V}} =\frac{{\int }_{0}^{t}{C}_{t}{Q}_{t}\Delta t}{{\int }_{0}^{t}{Q}_{t}\Delta t}$$

where *n* is the index or point in the storm event; *M* (g) is the pollutant mass during the rainfall event; *V* (m^3^) is the runoff volume during the rainfall event; C _*t*_ (mg/L) is the pollutant concentration at time *t*; Q _*t*_ (m^3^/s) is the discharge runoff flow rate at time (t); *t* refers to the time of total runoff; *t* _*i*_ is the time up to point *n* in the event, and *△t* is the interval time of sampling. Finally, *A* (km^2^) is the catchment area.

### Sensitivity analysis

Uncertainty in hydrological models arise from the inherent uncertainty of the model parameters. These parameters significantly influence stormwater simulations (flow peak and volume and pollutant load), hence a sensitivity analysis is crucial^33^. Ballinas-González et al.^40^ reported that peak time and peak flows are significantly affected by the length of flow compared to the soil moisture content. A relatively smaller N-impervious value can generate low runoff volume but a higher peak flow^40^. Liu and Chaubey^41^ reported that the CN can be the most sensitive variable, which may have significant effect on flow peaks. The sensitivity analysis is crucial for this study because it will help in providing a confidence level to the results derived from it. Besides, the slope of the selected watershed in this study ranging from 0.7 to 15%, can be considered a sensitive parameter. Surface depressions in the watershed are also crucial because it can increase storage capacity and eventually reduce peak and total flows. Also, reduction in percent zero imperviousness without any depression storage in the catchment causes reduction of peak flows. Again, Peak flows and time to peak were found to be significantly affected by the Manning coefficient^42,43^. Sensitivity of dynamic hydrological model is tested in the PCSWMM platform using rainfall-runoff simulations. Multiple hydrological and catchment related hydrological model parameters such as sub-catchment width, zero imperviousness, percent impervious, slope and curve number were considered for sensitivity analysis. Table 3 summarizes the parameters selected to investigate the sensitivity of the model. We tested the changes in the rainfall-runoff simulation results (e.g., peak flows) for changes in the selected parameters. We also use this testing strategy to optimize the parameters of the rainfall-runoff model.

**Table 3:** SWMM parameters for the sensitivity analysis.

Parameters	Values	Initial values source
Sub-catchment Width	Variable	Geometry
Percent imperviousess Percentage (n)	0.01–0.2	SWMM Manual
Percentage of impervious that has no depression (% Zero) Area	5–20	SWMM Manual
Slope	0.05–02	Topography and Surface Deformation (DEM)
Curve Number (CN)	Variable	King county and from the USDA Web Soil Survey

#### Model calibration

The PCSWMM uses a dynamic rainfall-runoff hydrological model to simulate stormwater runoff. The model is formulated based on different physical processes, which are embedded into the model through different equations defined with various parameters. The calibration in the PCSWMM is often performed by fine tuning of the parameters following the trial-and-error exercises with the objective function to reduce the errors between the observed and simulated flows. Hydrodynamic models are often calibrated by changing the model parameters to optimize the model performance. In this study, the model in the PCSWMM is calibrated against the observed flow rates and precipitation. Similar approach is also use by Abduljaleel and Demissie^18^ for the same study area to simulate stormwater runoff and find that imperviousness and depression storage are the most vital parameters for calibrating the model. Adjustments in sub-basin width, percentage imperviousness, curve number, and percentage zero storage were also considered for fine tuning the PCSWMM model. The Nash Sutcliffe Efficiency (NSE) statistics was used to assess the similarity between observed and simulated hydrographs in calibrating the model. In hydrological modeling NSE is often used for assessing the reduction of simulation errors during the calibration of the model. The NSE was evaluated as per Eq. (2)^44^:

2 $$NSE=1-\frac{\sum_{i=1}^{n}{\left({O}_{i}-{P}_{i}\right)}^{2}}{\sum_{i=1}^{n}{\left({O}_{i}\infty -\overline{O }\right)}^{2}}$$

where O and P are the observed and simulated flows, respectively. NSE statistics ranges from 1 to -∞; where 1 represents a perfect fit and a value of zero or less than zero indicates that the model is not better than the mean value. However, NSE is sometime strongly sensitive to peak flows (high or low) because it is computed as the squared differences of the observed and predicted flows^45^. Tan et al.^46^ compared the SWMM model calibration considering both continuous and event-based storms. Results revealed that both approaches simulate direct runoff volumes and hydrographs reasonably well but none of the approaches effectively simulated relatively lower runoff generated by low rainfall amounts. Due to its wide acceptance and application, NSE is also used in validating runoff simulations for watershed equipped with LIDs. Ahiablame et al.^47^ used Nash–Sutcliffe values for validating hydrodynamic model for simulating baseflow.

### Scenario development

**Figure 5:**
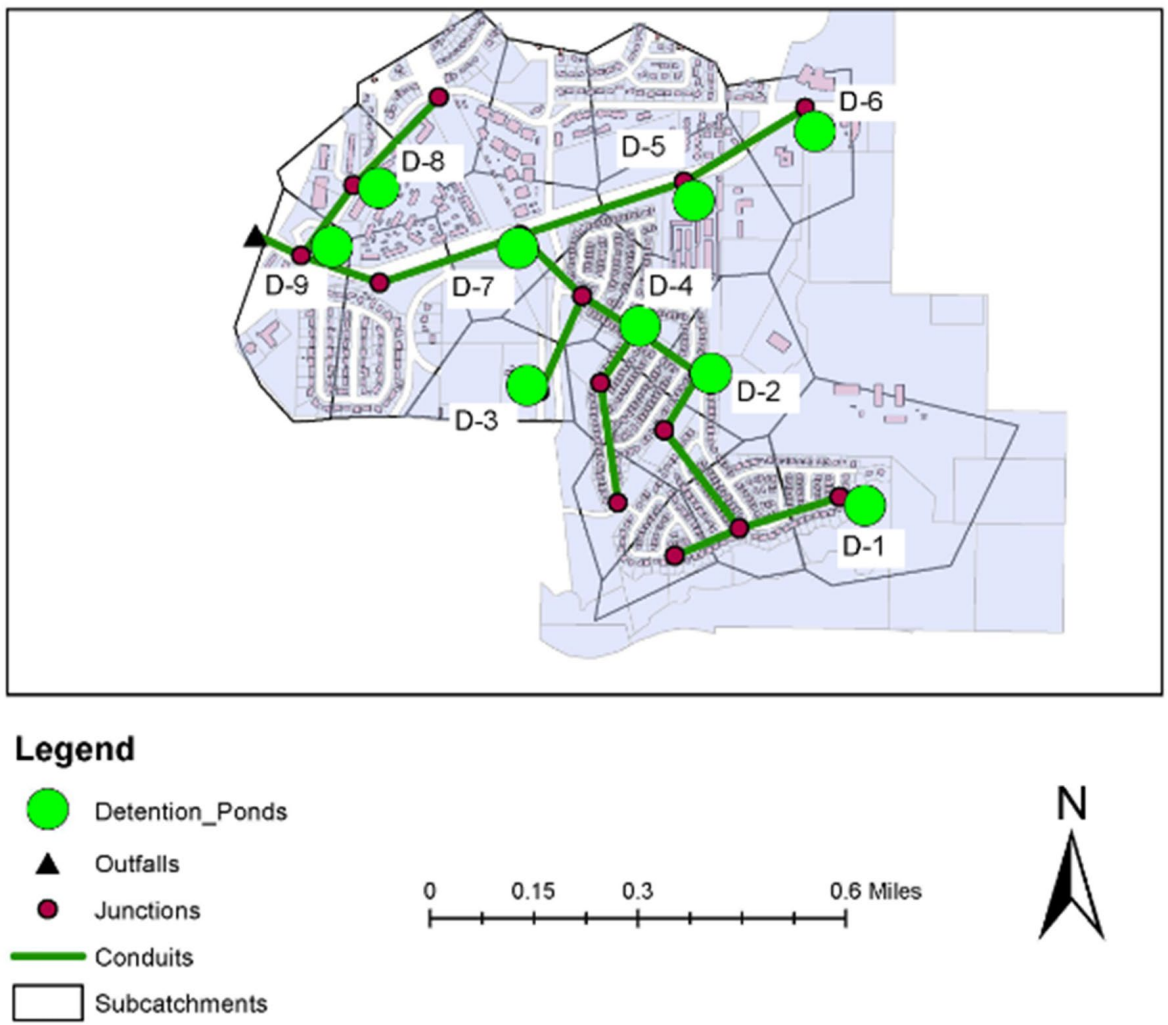
Drainage system in the Renton city watershed, with conduits, junction, outfalls, and sub-catchments.

As mentioned earlier, the existing stormwater system in the study area comprises a network of underground pipes, catch basins, curbs, and gutters that collect stormwater runoff throughout the watershed and direct it to a detention pond for retention and natural treatment. The system is composed of 24- and 36-inch-diameter helical corrugated metal pipes (HCMP) that collect runoff from the main subdivision and some undeveloped areas to the northwest of the watershed. The benchmark scenario is the conventional stormwater system without any detention pond, while the other scenarios include the proposed detention facilities in addition to the existing conventional stormwater system. The proposed volume of the detention ponds calculated by PCSWMM were modified by including the factor of safety between contours 3.5 m and 7 m for the safety requirements. The interior pond slopes were assumed to be graded with a 2:1 (H:V). The calibrated and validated hydrological model in the PCSWMM was adjusted with nine scenarios of detention ponds based on their size and locations over the historical and future periods (for all GCM-RCM models) to evaluate their effectiveness in reducing peak flow and pollutant loads (Fig. 5). Drainage regions were classified into three types based on each sub-catchment’s tributary drainage area, where detention ponds can be installed. Following that, the locations of these detention ponds were improved by categorizing them on the basis of soil types and runoff characteristics. The sizes of these detention ponds were selected based on the peak flow rate for a specific design storm (i.e., 100-year return period).

Nine detention ponds across the watershed were represented by the filled circles (green) (D-1 to D-9). Detention pond 1 (D-1) has an approximate capacity for runoff, and a pollutant load of 225 m^3^. Nearly 2.94 km^2^ of undeveloped upstream area drains to the D-1. In this detention pond, the daily pollutant loads of total nitrogen, total phosphorus, and TSS are approximately 7 kg, 4 kg, and 3950 kg, respectively. The stormwater drainage from the developed areas on the northern and southern parts covers lawns, roofs, and driveways of the residential lots, as well as asphalt and concrete pavements. D-2 is located at the north end of the trunk system and has an approximate capacity of 245 m^3^. D-3 and D-4 have an approximate capacity of 450 m^3^, located at the downstream of the trunk system. D-3, D-4, and D-6 were designed to capture stormwater draining from a heavily vegetated driveaway, along the eastern side of 120th Avenue at the intersection with 192nd Street. Also, the ponds receive runoff from an approximately 4.75 km^2^ area on the western side of the watershed. Evidence indicates that runoff flowed across the driveway, increasing total phosphorus, TSS, and phosphorus measured in that location, which were 3800–4500 kg and 3–4.5 kg, respectively. D-5 and D-6 have a capacity of 230 m^3^ and are located on the western side of the watershed (see Fig. 5). The D-7 is designed to collect storm runoff from the northwest part of the watershed having a capacity of 227 m^3^. D-2, D-5 and D-7 are designed to control peak flows by trapping runoff. The expected rate of pollutant collections of these detention ponds are 4–9 kg, 2–6 kg, and 3000–5000 kg for total nitrogen, total phosphorus, and total suspended solids, respectively. Detention ponds D-8 and D-9 have a capacity of 125 m^3^ are designed to capture storm runoff from the southeast. The proposed detention ponds consist of two treatment cells to provide both water quality treatment (through dead storage in the wet pool) and flow control (through live storage above the wet pool which will reduce peak flows to the outfall system and lower erosion and gullying at the downstream of the pond).

Additionally, the live storage in these detention ponds has a 3:1 side slope depending on the size of each pond. A safety factor of 1.2 as per the EPA recommendation was considered for live storage. The PCSWMM model calculated the peak rates and total mass of pollutant depending on characteristics of the basin (i.e., area, curve number (CN) and time of concentration (Tc)) and the stormwater conveyance system (e.g., pipe network and detention ponds). The modelled peak flows and volumes were used in the analysis of the existing drainage system and to size the flow control outlet structures and detention ponds.

## Results and discussions

### Calibration and validation

**Figure 6:**
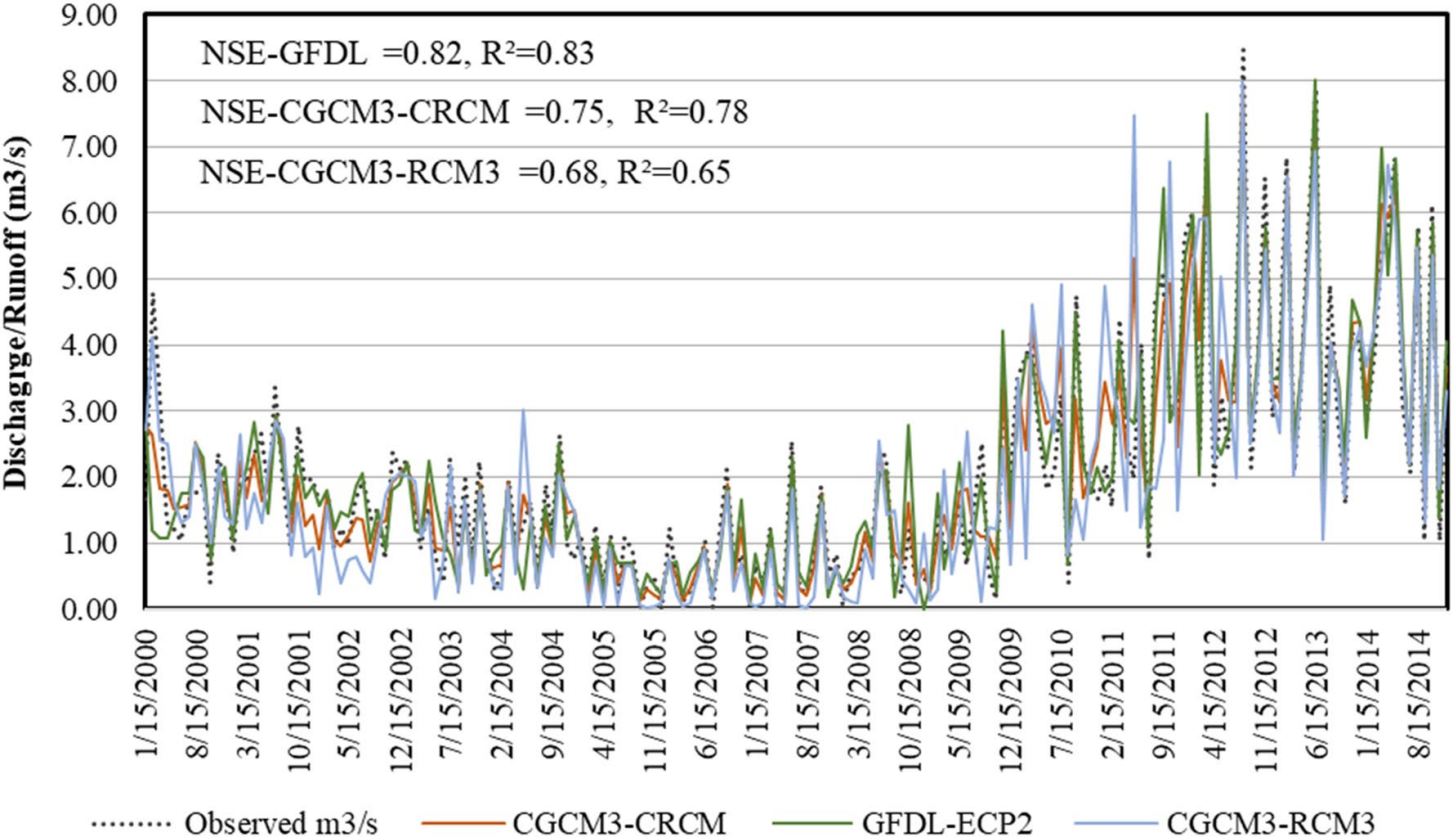
Comparison of modeled discharge with baseline (2000–2014).

Precipitation data from GFDL-ECP2, CGCM3-CRCM and CGCM3-CRCM climate models were tested to identify the best performing bias-corrected rainfall time series that accurately capture the observed variability. Figure 6 shows the time series of observed and bias-corrected daily precipitation for the GFDL-ECP2, CGCM3-CRCM and CMCC climate models. Plot shows that the bias-corrected precipitation well captures the overall variability of the observed precipitation. Efficiency statistics (i.e., NSE and R^2^) indicated that they are generally but the performance of the bias-corrected precipitation vary depending on the climate models. Based on the efficiency statistics the performance of the models can be ranked as GFDL > CCCM3-CRCM > CGCM3-RCM3.

**Figure 7:**
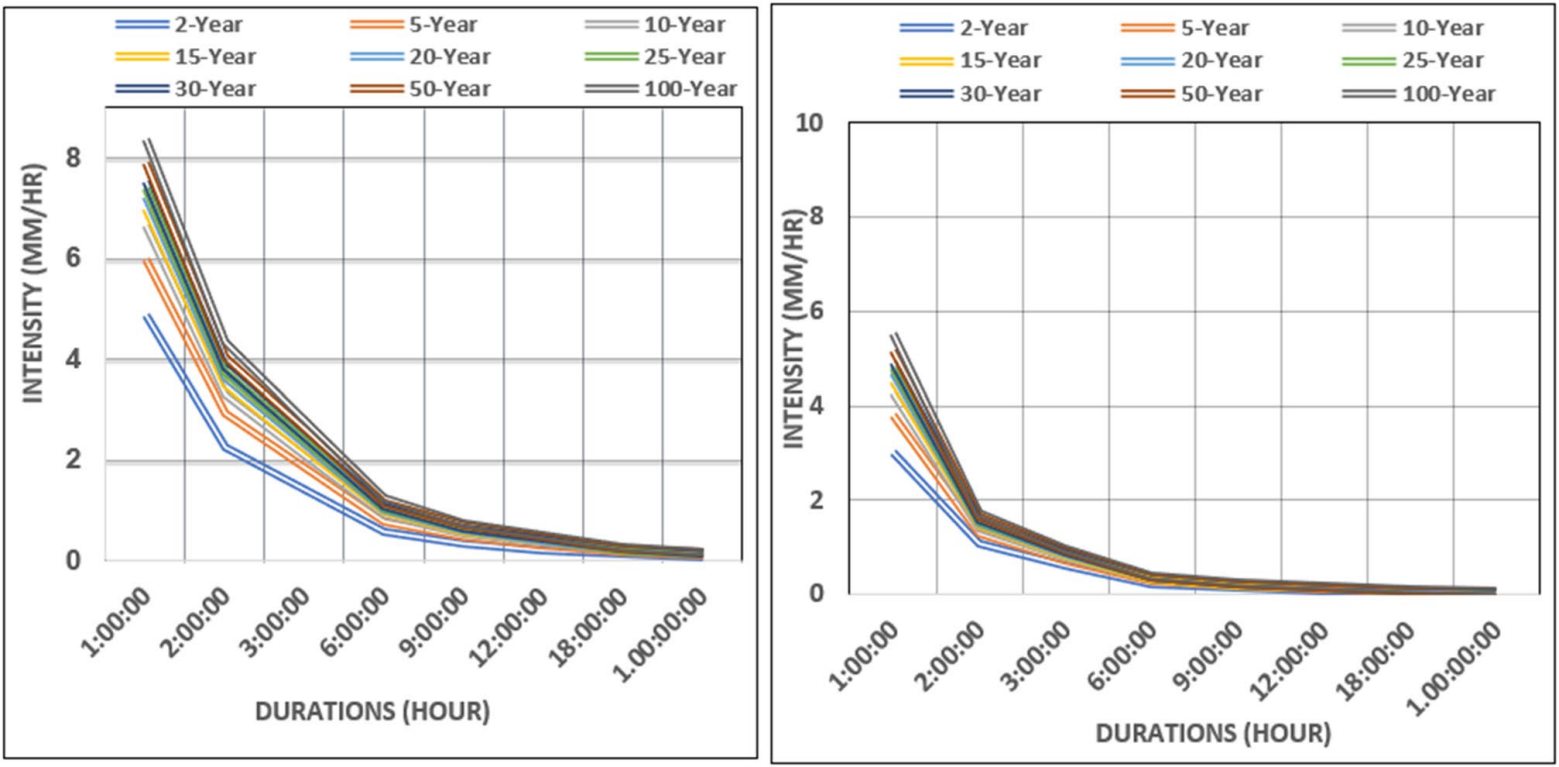
Comparison of intensity duration frequency (IDF) between historical and future scenario.

**Figure 8:**
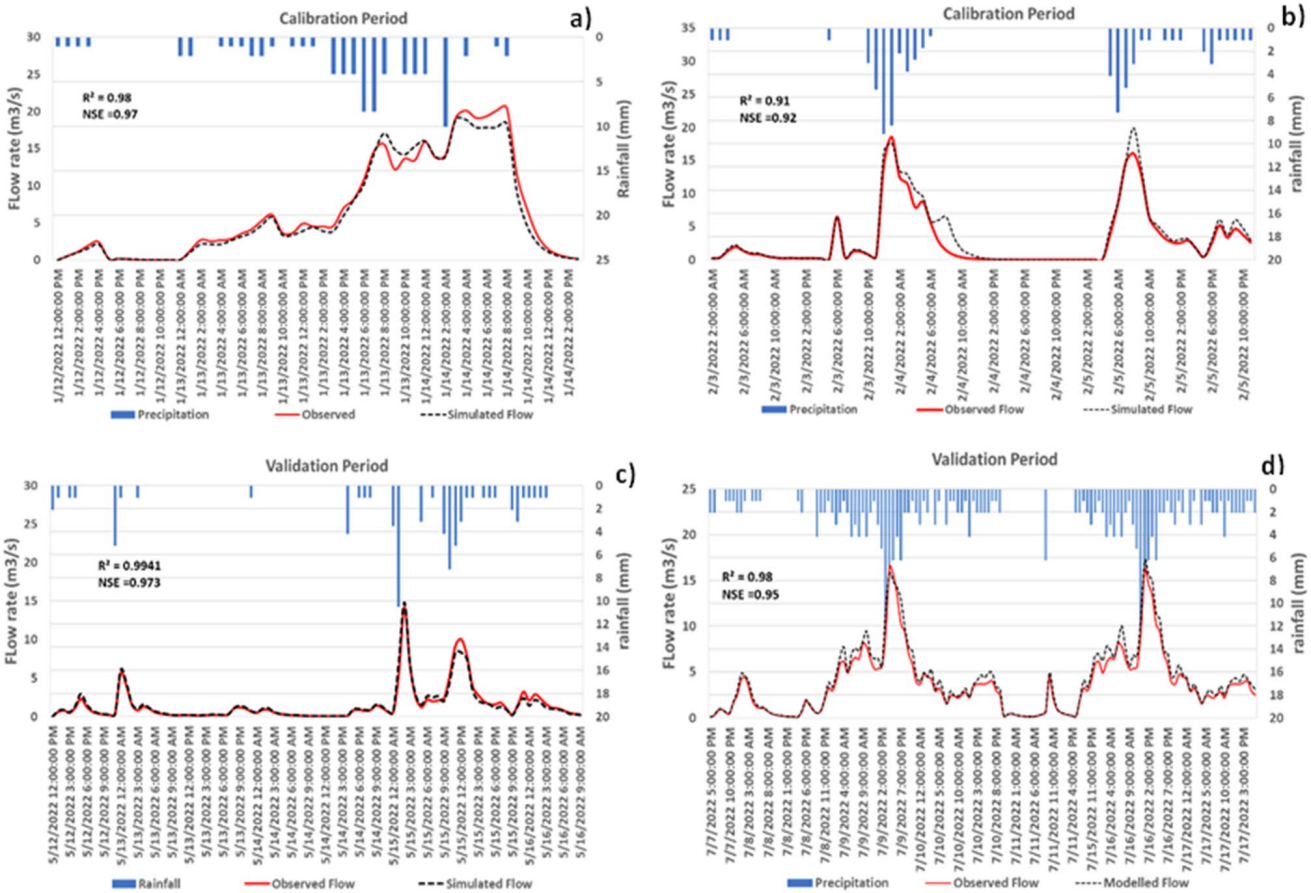
Model performance in simulating the observed flow hydrograph corresponding to the selected rainfall event (8th October 2021) for calibration and validation.

We developed rainfall Intensity–Duration–Frequency (IDF) curves for the future period using the future bias-corrected rainfall from the climate models. Mean rainfall averaged over the all-climate models were considered for developing IDF curves. Results indicate an increase 17 mm/h. In rainfall intensity is likely over 2023 to 2050 compared to the historical period 2000–2014. Also, almost all climate models indicate that there will be significant increase in the rainfall intensity; a 50-year storm events observed in the historical period is expected to be a 100-year event by 2050s (Fig. 7). For calibration and validation of the PCSWMM model, two separate storm events were considered. The results indicated that the model reasonably well reproduced the storm runoff for each storm events both in the calibration and validation period with reasonably high efficiency in terms of R^2^ and NSE as shown in Fig. 8.

**Figure 9:**
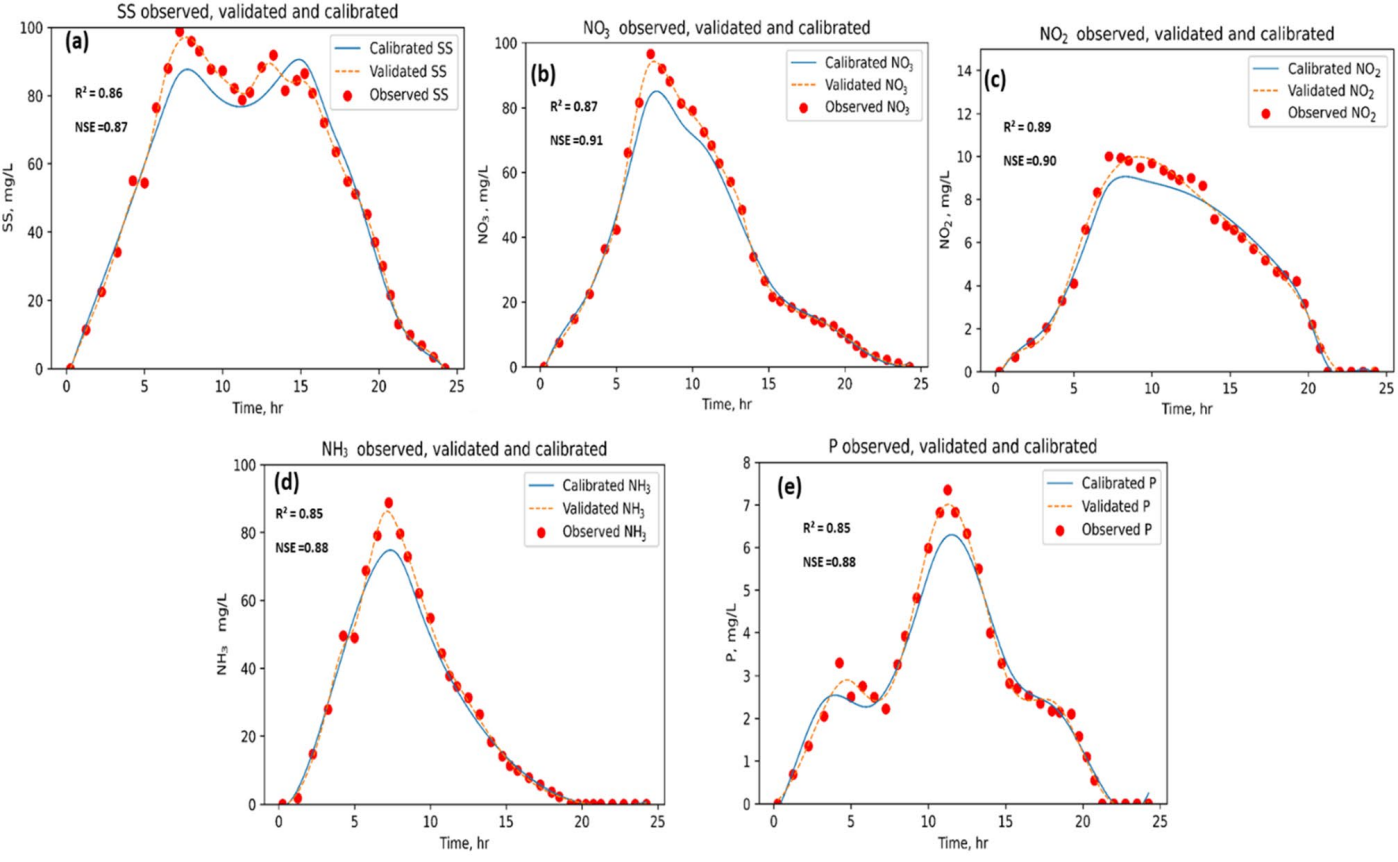
Calibration of the model for pollutant load during the selected event.

The study also examined the performance of the PCSWMM model to predict pollutant transfer load comparing the model prediction at a water quality monitoring station located at the outfall the catchments. Results showed that the model simulates pollutant load reasonably well, with the NSE statistics of 0.87, 0.91, 0.90, 0.88, and 0.88 for suspended solids (SS), nitrate (NO_3_), nitrite (NO_2_), ammonia (NH_3_), and total phosphorus (TP), respectively (Fig. 9).

**Figure 10:**
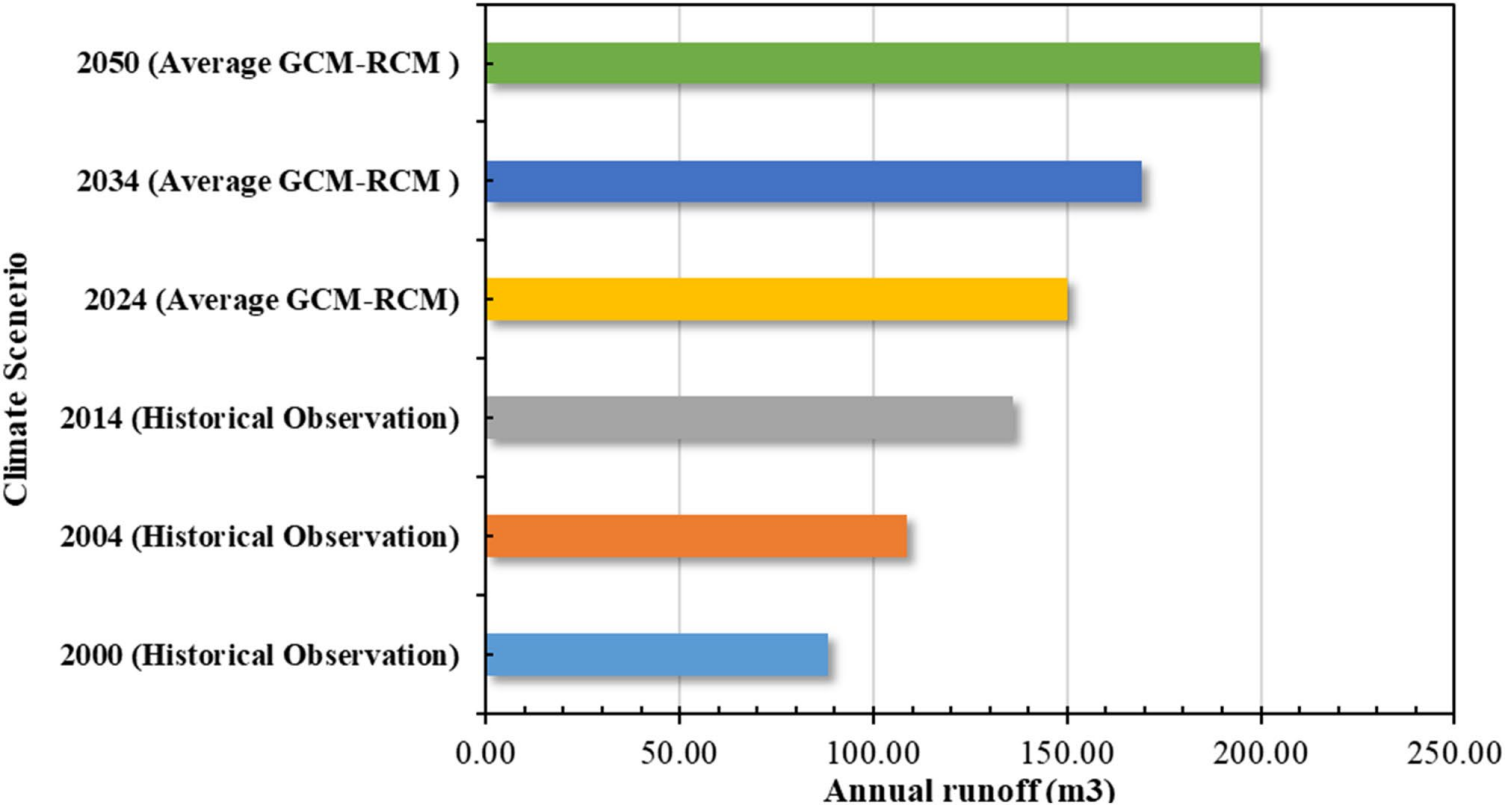
Annual runoff volume under climate change scenarios.

To investigate the climate change impacts on the stormwater generation which will eventually trigger the modification of existing stormwater management system and adopting new technologies, we quantified the future changes of stormwater runoff compared to the historical ones. Figure 10 represents the estimated annual storm runoff for different historical and future years. Rainfall averaged over the selected GCM-RCM was considered to quantify the mean annual storm runoff for the future period. The results indicated that the runoff volumes are likely to increase by up to 55% over the future periods (2024–2050) compared to the historical period.

### Evaluation of changes in pollutant load due to climate change

**Figure 11:**
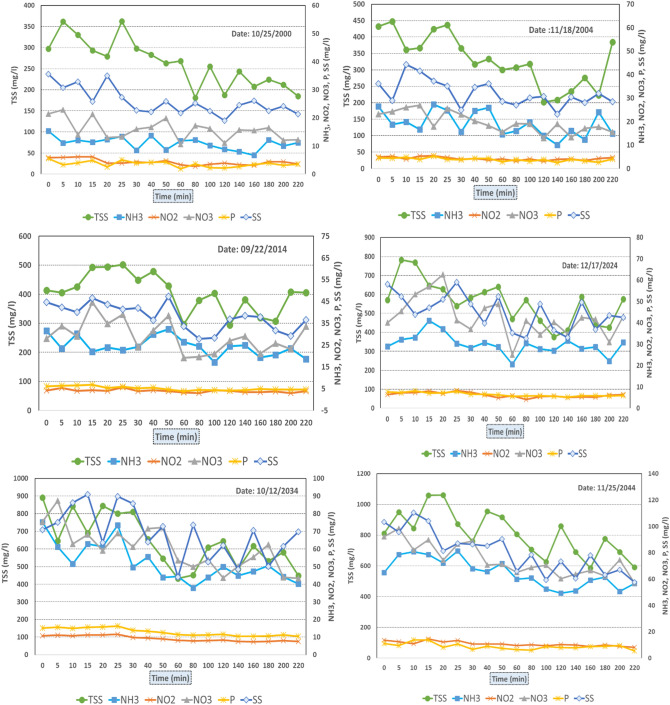
Simulated pollution runoff under climate change.

We quantified and compared the pollutant loads for the historical and future periods as illustrated in Fig. 11. As discussed in the methodology we estimated the pollutant loads using the total storm runoff. Results showed that the TSS is higher in 2014 than in 2004 due to increased runoff caused by increased rainfall intensity. Likewise, NH_3_ and NH_2_ increased slightly over the year 2004. Analysis indicated that the peak pollutant concentrations were higher than the historical observations for the 100-year storm events in the future climate compared to that of the historical events. During the historical event on 25/10/2024, the peak concentrations of TSS, SS, P, NO_3_, NO_2_, and NH_3_ are 789 mg/L, 681 mg/L, 10 mg/L, 705 mg/L, 9.4 mg/L and 465 mg/L, respectively. Increase in precipitation intensity and total runoff in the future climate generate higher pollutant load compared to the historical ones. For example, 100-year storm event under the future scenarios, the peaks of the total suspended solids (TSS) increased by 12.2%, to 27.5. Moreover, the results also indicated that the peak values of the pollutants were attained relatively quicker in the future compared to the historical ones due to faster rise in the future hydrograph, which was consistent with the study by Sharma et al. SpringerPlus (2016). Increases in the peak concentration of SS, P, NO_3_, NO_2_, and NH_3_ or future years (2024–2044) were identified as 28.4%, 16.6%, 15.2%, 22.4% and 48.9%, respectively. The stormwater pollution loads were likely to be larger under the warmer future climate due to the changes in flashy rainfall and storm runoff over a short period of time in the future.

### Sensitivity analysis

The results pertaining to the sensitivity analysis of variables for PCSWMM are given in Table 4. When the sub-catchment width was changed by (5–15%), the peak flows are reduced at a rate of 11.16–22.67%. Whereas, 13.10–21.53% reduction is observed due to changing the imperviousness by 5–15%. Zero percentage impervious area with no depression storage reduced peak flow from 12.28 to 19.83%. CN was identified as the most sensitive parameter having relatively higher influence in minimizing peak flows ranging from 12.29 to 22.74% for corresponding changes of CN from 5 to 15%. Other studies also identified CN as the most sensitive factor for water flows and quality, for example, Pereira Souza et al.^48^. In contrast, slope was the least sensitive parameter and it provided 8.65–14.9% reduction in peak flows.

**Table 4:** Sensitivity analysis results of SWMM model.

Parameter test	Percentage change (%)	Reduction in peak flow (%)	Percentage change (%)	Reduction in peak flow	Percentage change (%)	Reduction in peak flow (%)
Zero Percentage (% Zero)	5	− 12.28	10	− 15.6	15	− 19.83
Sub-catchment Width	5	− 11.16	10	− 13.51	15	− 22.67
Imperviousness (n)	5	− 13.10	10	− 17.45	15	− 21.53
Curve Number (CN)	5	− 12.29	10	− 16.98	15	− 22.74
Slope	5	− 8.65	10	− 11.23	15	− 14.90

### Impact of detention ponds facility on peak flow

**Figure 12:**
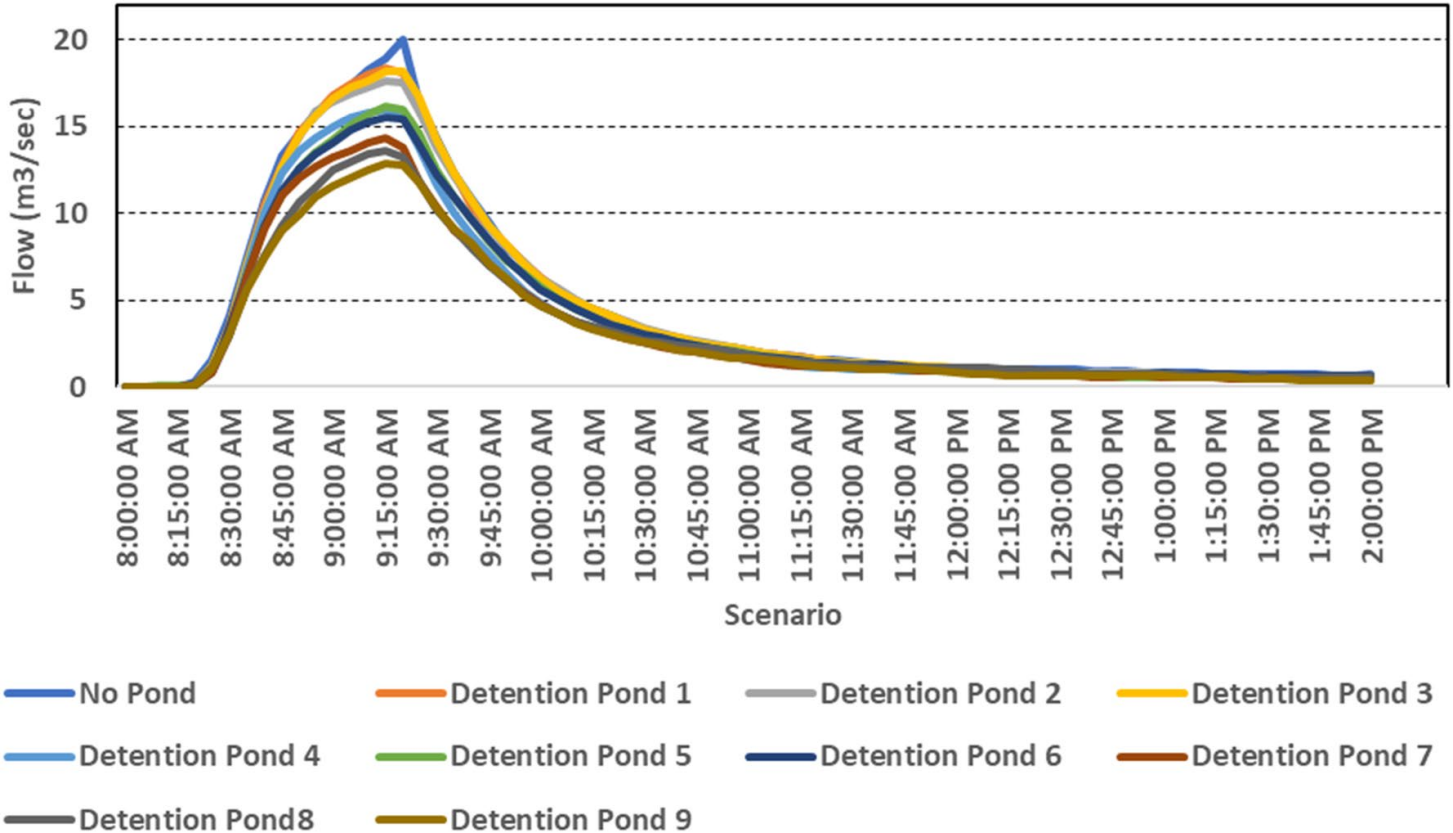
Hydrographs showing flow reduction when different detention ponds were considered compared to the base scenarios without detention pond.

The simulated flows corresponded to the scenarios with and without the suggested detention pond systems in addition to the existing stormwater system (Fig. 12). The results demonstrated that, when compared to the conventional stormwater management system without detention ponds, the proposed detention ponds led to significant flow rate reductions. However, reduction in peak flow rate varied depending on the selected detention ponds. D-7, D-8, and D-9 were the best in terms of mitigating the flows to the outfall; among them, D-9 has the highest flow reduction rate (28.7%), compared to the benchmark scenario (without detention ponds). D-1, D-2, and D-3 had the least effects on the flow rate reduction; D-1 showed the smallest decline in the flow rates (i.e., 2.8%). D-4, D-5, and D-6 performed slightly better in reducing the peak flow rates. Overall, the results indicated that the implementation of detention ponds had a potential to reduce urban flooding risk caused by the increased storm intensity in the future. The selected design parameters of these detention ponds varied in terms of depth and residence time, ranging from shallow detention ponds (1.2–2.8 meter-deep) with short residence times (14 h during the rainy season) to deep detention ponds (1.5–3.2 meter-deep) with longer residence times (> 24 h). The design parameters and the location of the detention ponds provided variable results in reducing peak flows.

### Pollutant load reduction

**Figure 13:**
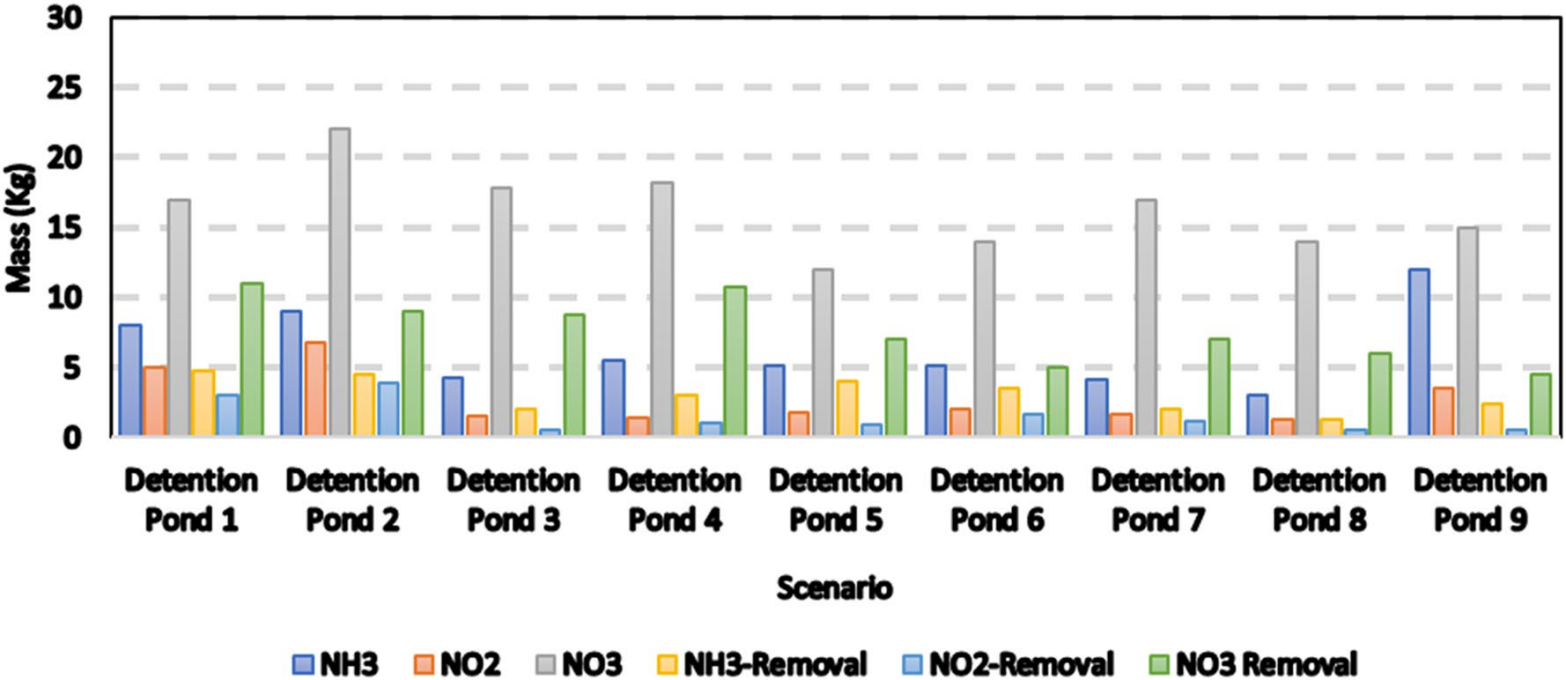
Percent removal of NH_3_, NO_2_, and NO_3_ in the storm runoff for different scenarios of detention ponds.

Figure 13 shows the reduction of various pollutant loads for scenarios corresponding to different detention ponds compared to the base scenario where no detention ponds are considered. Simulation results show that D-8 and D-9 are the most effective in reducing pollutant transport. The pollutant reduction rates of D-9 were 57%, 60%, and 75% for NH_3_, NO_2_, and NO_3_, respectively whereas the reduction rates obtained from D-9 are 80% 86%, and 70%. The high penetration rates, in addition to the high surface storage capacities of the detention ponds, are the most prominent factors in achieving significant reductions. D- 6 and D-1 provide the lowest reductions in NO_2_ (18%) and NO_3_ (35%), whereas D- 5 shows the lowest drop in NH_3_ load (23%). Analysis indicates that increasing the slope to divert water and controlling the size of inlet will help to delay runoff. Also, the detention ponds can naturally enhance water quality with the natural vegetation around the ponds.

**Figure 14:**
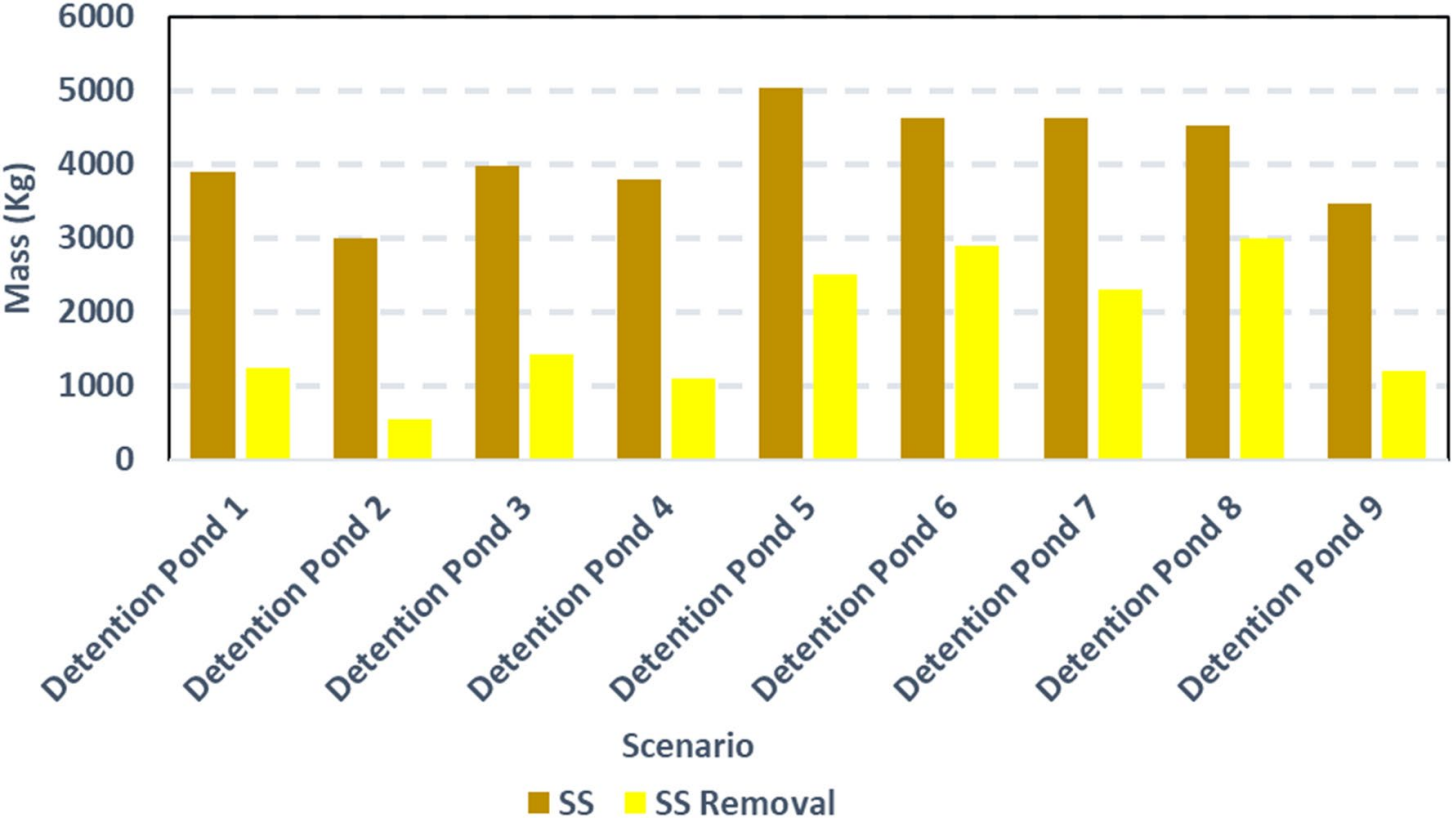
Simulations of Suspended Solid removal as a result of considering the implementation of detention ponds.

As previously stated, the location and topography of the detention ponds affect the reduction in flow rates and concentration of pollutants in the storm runoff. 81% of the suspended solid load was reduced by detention pond 2, while detention pond 8 caused the lowest (34%) reduction in suspended loads (Fig. 14). The sedimentation potential of these detention ponds is influenced by four major factors: soil characteristics, vegetative cover, topography, and microclimate. Sedimentation control is achieved by a combination of structural measures, cover measures, and construction practices that are tailored to fit the specific location.

**Figure 15:**
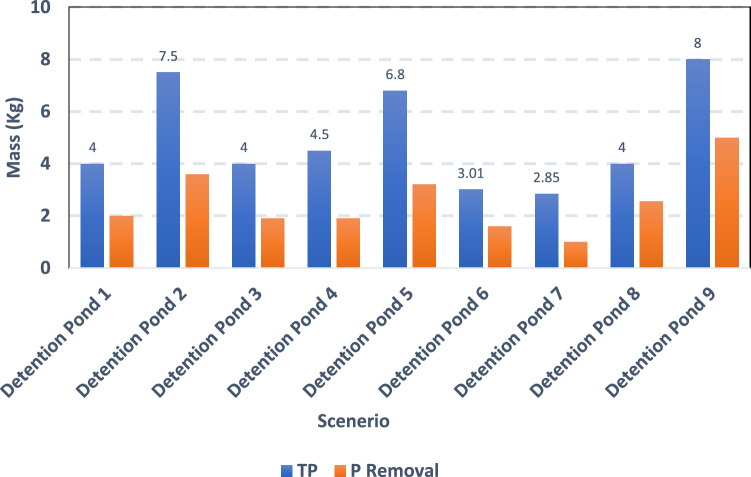
Removal (in kg) of total phosphorite (TP) and phosphorite (P) for different scenarios of detention ponds.

The drainage of phosphorus from urban areas and lawns has already significantly impacted some waterbodies of the city. The locations of the detention ponds were chosen depending on the residential area to trap large concentrations of phosphorus and allow these particulates to settle in the sediments. Figures 14 and 15 depict how each detention pond reduced phosphorus in the storm runoff when compared to the baseline situation (without detention ponds). The results indicated that when the proposed detention ponds were taken into consideration, reduction in phosphorus concentration in terms of total phosphorus was significantly high (up to 65%).

## Conclusion

Stormwater management is a critical task for urban environments, getting more challenging with climate changes. Future increase in the frequency and intensity of precipitation as suggested by different climate models adequate and appropriate technologies should be tested and integrated to the conventional stormwater management system to reduce the urban flood risks and increased pollutant load. The present study investigated the effectiveness of a green stormwater infrastructure option (i.e., detention ponds) in minimizing the flood risks by lowering peak flows and reducing pollutant loads. The stormwater model was calibrated and validated for the watershed located in the City of Renton, on the southern shore of Lake Washington, USA, using the PCSWMM modeling framework. The model performed well with high model efficiency (R^2^ of 0.93 and 0.97 and NSE of 0.86 and 0.88 for the calibration and validation periods, respectively). With the trained model in PCSWMM, the study assessed the performance of different detention ponds (variable in size and locations) in reducing peak flows and total pollutant loads across the watershed. The study quantified and compared the simulation results considering the detention ponds to the base scenario where no detention ponds were considered to assess the positive impacts of detention pond in reducing peak flows and pollutant loads with the target if these could be available options in urban stormwater management for combating future stress of climate change related increase in rainfall frequency and intensity. Different sizes of detention ponds were tested in the study. It was revealed that detention ponds are capable to significantly reduce the flow rate when compared to the scenario without detention ponds. Analysis indicated that the performance of the stormwater management system with detention ponds varied depending on the size and location of the selected detention ponds. We also showed that the detention ponds can be adopted to reduce the climate change impacts on stormwater management in urban watershed.

To investigate the future changes of storm runoff and corresponding pollutant loads due to climate change related increase in the frequency and intensity of precipitation, we adjusted the calibrated stormwater water model with the future precipitation from different climate models in the PCSWMM platform. We found that stormwater runoff and pollutant loads are likely to increase significantly in future, indicating the necessities of adopting innovative infrastructures. Results showed that the stormwater runoff is expected to increase by 58.4% for the future period 2023 to 2050 for GFDL-RCM3 model scenario compared to the historical period. We simulated if detention ponds can be integrated into the existing stormwater system to mitigate the future challenges of urban flood risk and pollutant loads using 10 different scenarios of detention ponds formalized based on their sizes and locations. Results depicted that the installation of detention ponds could significantly reduce the stormwater runoff (up to 28.7%) compared to the baseline condition (in which no detention pond was considered). Significantly high reduction in the pollution loads were evident when detention ponds were integrated to the existing stormwater management system. It is observed that soil penetration rates and the land's potential for surface storage have a significant impact on restricting the movement of pollutants, though vary for different detention ponds due to their sizes and locations. It was established that the detention ponds can remove the pollution load up to 80%, 86%, and 80% for NH_3_, NO_2_, and NO_3_, respectively.

The findings of the study showed that installation of detention ponds is a promising option to mitigate the adverse impacts of changed storm events in the future climate. However, the study has a few limitations, for example, the study considers only detention pond as an option while other low impact development (LID) options and their combinations could also be tested in minimizing flood risk and pollutant loads. Moreover, the study focused on some promising pollutants such NH_3_, NO_2_, and NO_3_, suspended solids, and total phosphates, other emerging pollutants such as polycyclic aromatic hydrocarbon (PAH) could be considered. Though the emerging pollutants are challenging to simulate, they could be considered for future study. We considered each detention ponds separately to assess their performance, however, optimization algorithms (e.g., genetic algorithms) could be employed to optimize the detention in terms of size, total number, and implementation locations. Though this was not the focus of the present study, we suggested an optimization exercise for the future study. Overall, it has been found in the study that detention pond could be used for coping with future climate challenges in stormwater management by minimizing stormwater runoff and corresponding pollutant loads. The framework for investigating the potential of different green infrastructure for climate change adaptation in the stormwater management sector, is transferable and can be applied to other regions.

## Data Availability

The datasets used and/or analyzed during the current study available from the corresponding author on reasonable request.
